# A case of late-onset *Klebsiella oxytoca* keratitis treated with topical imipenem after deep anterior lamellar keratoplasty

**DOI:** 10.5935/0004-2749.20210043

**Published:** 2021

**Authors:** Özer Dursun, Erdem Dinç, Ömer Özer, Şeyma Kıroğlu, Mustafa Vatansever, Ufuk Adıgüzel

**Affiliations:** 1 Department of Ophthalmology, Faculty of Medicine, Mersin University, Mersin, Turkey

**Keywords:** Corneal transplantation, Lamellar keratoplasty, *Klebsiella oxytoca*, Keratitis, Imipenem, Transplante e córnea, Ceratoplastia lamelar, *Klebsiella oxytoca*, Ceratite, Imipenem

## Abstract

The aim of this study was to discuss a case of late-onset *Klebsiella oxytoca* keratitis after deep anterior lamellar keratoplasty and its treatment. A 21-year-old female patient presented with redness and effluence in the left eye at 5 months after uncomplicated deep anterior lamellar keratoplasty surgery. In the examination, a single suture was loosened in the superior nasal region and there was an infiltration area and epithelial defect in the graft and recipient bed junction in the area of the loose suture. Topical fortified vancomycin and fortified ceftazidime treatment was started empirically hourly, but there was insufficient response. After *K. Oxytoca* growth in a swab and suture culture taken from the patient, fortified vancomycin was replaced with fortified imipenem. It was observed that the infiltration area rapidly regressed and the epithelial defect was closed after fortified imipenem treatment. Fortified imipenem may be considered as an alternative treatment, especially in cases in which there is no response to treatment and culture growth is detected.

## Abstract (pt)

O objetivo deste estudo é discutir um caso de ceratite tardia por *Klebsiella oxytoca,* após ceratoplastia lamelar anterior profunda, bem como seu tratamento. Uma paciente de 21 anos apresentou vermelhidão e efluxo no olho esquerdo 5 meses após cirurgia de ceratoplastia lamelar anterior profunda sem complicações. Ao exame, havia uma única sutura solta na região nasal superior e uma área de infiltração com defeito epitelial no enxerto e na junção com o leito receptor na área da sutura solta. Iniciou-se empiricamente um tratamento tópico com vancomicina e ceftazidima fortificada de hora em hora, porém com resposta insuficiente. Após o crescimento de *K. oxytoca* a partir de cultura de *swab* e sutura retirados da paciente, a vancomicina fortificada foi substituída por imipenem fortificado. Observou-se que a área de infiltração regrediu rapidamente e que o defeito epitelial foi fechado com o tratamento com imipenem fortificado. O imipenem fortificado pode ser considerado um tratamento alternativo, especialmente nos casos sem resposta ao tratamento e detecção de crescimento na cultura.

## INTRODUCTION

Infectious keratitis is one of the most important complications of penetrating keratoplasty and poorly affects the success of the graft and visual outcomes^(1,2)^. The incidence of infectious keratitis after keratoplasty is reported to be between 1.76% and 7.4% in developed countries, while it can reach up to 11.9% in developing countries^(3)^. Infectious keratitis usually occurs in the first year after keratoplasty; in particular, suture-related problems are an important cause of this complication. Loose or exposed sutures disrupt the integrity of the epithelium in their region, and mucus strands attached to this region form a nidus for bacterial colonization, creating a serious predisposition^(1)^.

The most common cause of infectious keratitis after keratoplasty is Gram-positive cocci. In particular, *Streptococcus* species and coagulase-negative *Staphylococcus* species are the most common microorganisms causing graft infection^(1-2,4-5)^. *Pseudomonas aeruginosa* from Gram-negative microorganisms and *Aspergillus* species from fungal agents are among other important causes of this condition. *Klebsiella* species are Gram-negative microorganisms that are commonly found in nature and can be present among the gastrointestinal and nasopharyngeal flora under normal conditions. The main species that cause infection in humans are *K. Pneumonia* and *K. Oxytoca*, most commonly causing respiratory system, urinary system, biliary system, and surgical wound infections^(6)^. Moreover, they have rarely been reported to cause ocular infections^(7,8)^. The purpose of this study was to discuss a case of late-onset *K. Oxytoca* keratitis after deep anterior lamellar keratoplasty (DALK) and its treatment.

## CASE REPORT

**Figure 1:**
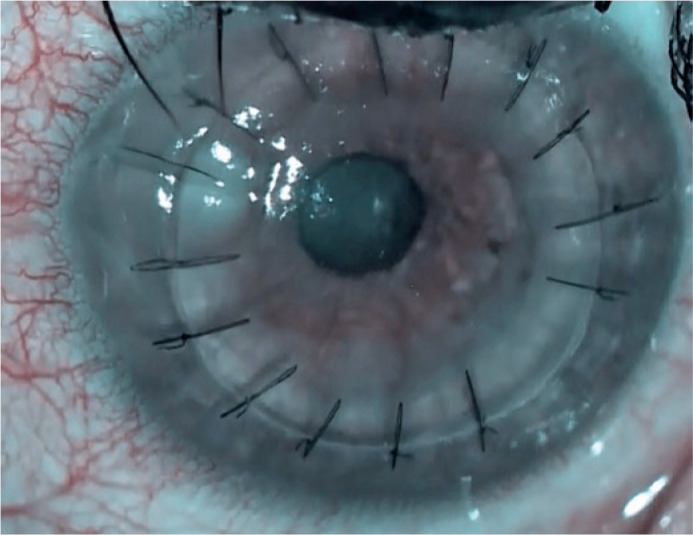
Infiltration area in the superior nasal region of the graft and recipient bed junction.

**Figure 2:**
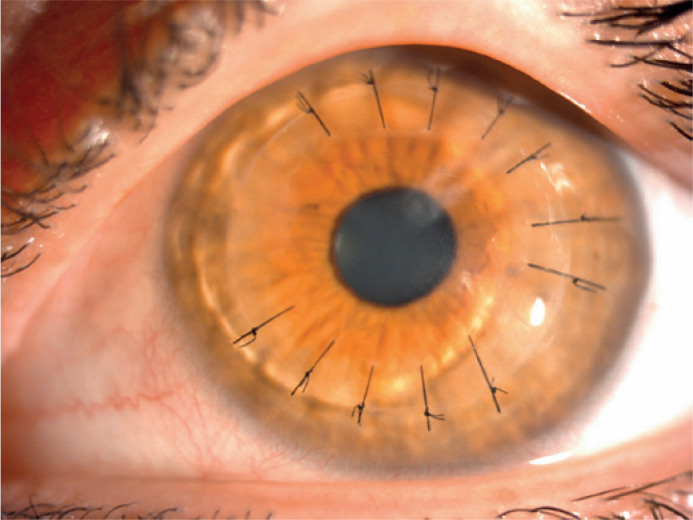
Healing of the infiltration area.

A 21-year-old female patient was admitted to our clinic with visual loss in both eyes. The patient’s history and family history were unremarkable. In addition, she had no history of trauma. The best corrected visual acuity (BCVA) was 5/10 in the right eye and 1/10 in the left eye. Anterior segment examination revealed bilateral Vogt striae, Fleischer ring, and corneal thinning, whereas posterior segment examination was normal. The patient was diagnosed with keratoconus in light of the examination findings and topography data, and cross-linking treatment to the right eye and DALK surgery to the left eye were performed at different times without any complication. BCVA improved to 4/10 after surgery in the left eye. However, she was admitted to the clinic with redness and discharge in her left eye at the postoperative 5th month. Visual acuity decreased to 1/10, a single suture was loosened in the superior nasal region and an infiltration area and epithelial defect were found in the graft and recipient bed junction, at the site of the loose suture (Figure 1). Suture-related keratitis was considered and the patient was hospitalized urgently. Swab samples were taken from the infiltration area and cultured at the bedside. Moreover, the suture was removed and sent for culture with the transport media. fortified ceftazidime (50 mg/ml), and artificial tears without preservatives were started every hour, and cyclopentolate was administered 3 times per day. Otherwise, the dose of loteprednol, which was started after keratoplasty, was revised twice daily. On the fourth day of treatment, there was no improvement in the in filtration area and epithelial defect, and *K. Oxytoca* growth was detected in both swab and suture cultures. An antibiogram revealed that the microorganism was susceptible to imipenem, and it was thought that there was insufficient response to the treatment. Therefore, fortified vancomycin was stopped and fortified imipenem (5 mg/ml) was added to the patient’s treatment. On the second day of fortified imipenem treatment, the infiltration area began to shrink, and the epithelial defect had closed completely on the 7th day. After epithelial closure, the loteprednol dose was revised four times a day. Fortified drops were discontinued within 1 month, and treatment was continued with preservative-free artificial tears and loteprednol. At the 6-month follow-up, her visual acuity was 6/10 and the infiltration area was completely healed (Figure 2).

## DISCUSSION

The main risk factors for the development of infectious keratitis after keratoplasty are suture-related problems, contact lens wearing, dry eye, acne rosacea, blepharitis, and poor hygiene conditions^(3)^. Although keratitis develops in the early period because of intraoperative contamination and infected donor cornea, late keratitis is usually associated with environmental contamination. The rates of infectious keratitis observed after lamellar keratoplasty are similar to those of penetrating keratoplasty and the risk factors have been reported to be similar. In the presented case, infectious keratitis developed because of suture loosening after DALK and, considering the causative agent, environmental contamination, and poor hygiene conditions may be considered to be effective in this table.

In the case presented here, loosening of the suture after DALK, environmental contamination, and poor hygiene conditions may cause infectious keratitis.

In recent years, *K. Oxytoca* has been found to cause more frequent nosocomial infections^(9)^. *K. Oxytoca* infection is mostly caused by non-ocular infections, such as pneumonia, antibiotic-related hemorrhagic colitis, pneumonia, and urinary tract and skin infections. This microorganism, the virulence of which is still not fully understood, leads to more frequent infections in cases of normal flora degradation and immunosuppression^(9)^. Rarely, various ocular infections may be caused by this microorganism. Late-onset corneal ulcer from flap margin after LASIK, and infectious keratitis after penetrating keratoplasty and endogenous endophthalmitis have been reported in the literature^(9)^. In our case, late-onset *K. Oxytoca* keratitis was found after DALK and, to the best of our knowledge, this is the first case reported in the literature to have occurred after DALK.

*K. Oxytoca* is one of the microorganisms that exhibit widespread antibiotic resistance. In the case presented here, broad-spectrum topical antibiotic treatment was started empirically after the culture, but there was insuffi cient response to this treatment. As a result of culture and antibiogram, treatment was re-arranged, and topical imipenem treatment yielded rapid recovery. In the literature, topical imipenem treatment has been reported to be very successful in multidrug-resistant *Pseudomonas Aeruginosa* keratitis^(10)^. This case showed that topical imipenem treatment may be an alternative in a similar case. In cases that do not respond to empirical treatment, topical imipenem treatment should be considered based on the antibiogram result. However, the development of drug resistance is an important point to keep in mind and it seems more reasonable to reserve imipenem as an alternative option.

In conclusion, *K. Oxytoca* may rarely cause ocular infections after surgery. Fortified imipenem may be considered as an alternative treatment, especially in cases in which there is no response to treatment and culture growth is detected. In similar cases, it is very important that appropriate microbiological sampling and an appropriate treatment protocol be started immediately. Hereby, good results can be obtained in terms of visual acuity.

## References

[1] Akova YA, Onat M, Koc F, Nurozler A, Duman S. (1999). Microbial keratitis following penetrating
keratoplasty. Ophthalmic Surg Lasers.

[2] Al-Hazzaa SA, Tabbara KF. (1988). Bacterial keratitis after penetrating
keratoplasty. Ophthalmology.

[3] Vajpayee RB, Sharma N, Sinha R, Agarwal T, Singhvi A. (2007). Infectious keratitis following keratoplasty. Surv Ophthalmol.

[4] Bates AK, Kirkness CM, Ficker LA, Steele AD, Rice NS. (1990). Microbial keratitis after penetrating
keratoplasty. Eye (Lond).

[5] Tseng SH, Ling KC. (1995). Late microbial keratitis after corneal
transplantation. Cornea.

[6] Kim BN, Ryu J, Kim YS, Woo JH. (2002). Retrospective analysis of clinical and microbiological aspects of
Klebsiella oxytoca bacteremia over a 10-year period. Eur J Clin Microbiol Infect Dis.

[7] Yeung SN, Lichtinger A, Kim P, Amiran MD, Slomovic AR. (2011). Late-onset Klebsiella oxytoca flap-margin-related corneal ulcer
following laser in situ keratomileusis. J Cataract Refract Surg.

[8] Sharma N, Gupta V, Vanathi M, Agarwal T, Vajpayee RB, Satpathy G. (2004). Microbial keratitis following lamellar
keratoplasty. Cornea.

[9] Wong JS, Chan TK, Lee HM, Chee SP. (2000). Endogenous bacterial endophthalmitis: an east Asian experience
and a reappraisal of a severe ocular affliction. Ophthalmology.

[10] Chatterjee S, Agrawal D. (2016). Multi-drug resistant Pseudomonas aeruginosa keratitis and its
effective treatment with topical colistimethate. Indian J Ophthalmol.

